# Role of Insulin and Growth Hormone/Insulin-Like Growth Factor-I Signaling in Lifespan Extension: Rodent Longevity Models for Studying Aging and Calorie Restriction

**DOI:** 10.2174/138920207783591726

**Published:** 2007-11

**Authors:** T Chiba, H Yamaza, I Shimokawa

**Affiliations:** Department of Investigative Pathology, Nagasaki University Graduate School of Biomedical Sciences, Nagasaki 852-8523, Japan

**Keywords:** Insulin, GH, IGF-I, calorie restriction.

## Abstract

Insulin/insulin-like growth factor-I (IGF-I) pathways are recognized as critical signaling pathways involved in the control of lifespans in lower organisms to mammals. Caloric restriction (CR) reduces plasma concentration of insulin, growth hormone (GH), and IGF-I. CR retards various age-dependent disorders such as nuerodegenerative diseases and extends lifespan in laboratory rodents. These beneficial effects of CR are partly mimicked in spontaneous or genetically engineered rodent models of reduced insulin and GH/IGF-I axis. Most of these long-living rodents show increased insulin sensitivity; however, recent study has revealed that some other rodents show normal or reduced insulin sensitivity. Thus, increased insulin sensitivity might be not prerequisite for lifespan extension in insulin/GH/IGF-I altered longevity rodent models. These results highlighted that, for lifespan extension, the intracellular signaling molecules of insulin/GH/IGF-I pathways might be more important than actual peripheral or systemic insulin action.

## INTRODUCTION

Compared to animals allowed to access food freely, moderate restriction of food intake, but not malnutrition, reduces morbidity and mortality in laboratory animals [1]. This effect, often noted as the anti-aging effects of caloric restriction (CR), has been recognized as a result from the restriction of calorie intake, but not from the restriction of a specific food component and not from a reduction of the toxic contaminants in food [2,3]. From an evolutionary viewpoint, the effect of CR appears to be explained by organisms having evolved adaptation mechanisms in their neuroendocrine systems to maximize survival during periods of food shortage [4]. Thus, it is believed that CR can, at least in part, regulate the aging processes through its effects on endocrine and/or neural regulatory systems [2]. The underlying molecular mechanism of regulation of the neuroendocrine system by CR remains to be fully elucidated; however, insulin, the GH/IGF-I axis, and leptin have been proposed as potential molecular mediators of the adaptive response to CR [5-8]. 

In many rodents, spontaneously or targeted gene modifications of insulin and the GH/IGF-I axis results in lifespan extension (see review [9-12]). Thus CR and these rodent insulin/GH/IGF-I models share, at least in part, a molecular mechanism of longevity. However, CR has been shown to also extend lifespan in GH-deficient long-lived Ames dwarf mice [13]. Furthermore, we found that CR significantly extended the lifespan of pituitary specific GH-antisense transgenic (TG) rats, which have reduced plasma levels of insulin and IGF-I [14,15]. These findings suggest that there might be different pathways in lifespan extension by GH/IGF-I suppression and CR; although it is possible that they share some of the same regulatory molecules in the insulin/GH/IGF-I pathway.

In lower organisms, long-lived mutants in *C. elegans* have provided a body of evidence about molecular mechanisms underlying longevity [16]. The *C. elegans* neurosecretory signaling system regulates whether animals enter the reproductive life cycle or have an arrest in development at the long-lived dauer diapause stage. The *daf-2* gene encodes an insulin/IGF-I like receptor, a critical gene in the genetic pathway that mediates this endocrine signaling in *C. elegans* [17]. The *age-1* gene [18] acts in the same signaling pathway as *daf-2*, and is closely related to a family of mammalian phosphatidylinositol 3-kinase (PI3K) p110 catalytic subunits under the insulin/IGF-I signaling pathway [19]. It has been also reported that mutations in the *Drosophila* equivalents of *daf-2* (*InR*), and *chico*, a fruit fly insulin receptor substrate (IRS), indicate that these genes have roles in body and organ size determination as well as in lifespan [20,21]. Reduction of function in *InR* mutants and the null mutation of *chico* increased the mean lifespan in female flies. These findings suggest that insulin/IGF-I signaling is a highly conserved longevity signal from lower to higher organisms.

In this brief review, we first discuss longevity rodent models that have modified insulin/GH/IGF-I pathways. These include spontaneous dwarf mutations and genetically altered rodents with increased lifespan. Later, we speculate the involvement of WD-repeat protein 6 (WDR6) as a candidate for the regulator of insulin/IGF-I signaling, which controls metabolism and longevity in the brain [22].

### Insulin and GH/IGF-I Axis in Rodent Longevity Models

Table **1** shows rodent longevity models with modified insulin/GH/IGF-I signals. In mammals, the production of IGF-I is stimulated by pituitary GH. *Prop-1* (Ames mice) and *pit-1* (Snell mice) mutant dwarf mice live longer than controls, with defects of transcription factors controlling differentiation of the anterior pituitary during fetal development [23]. These mice are also prolactin (PRL) and thyroid-stimulating hormone (TSH) deficient. However, it is thought that inhibition of the GH/IGF-I axis is the primary effector on lifespan extension of these mice because GH overexpression mice show resemble an accelerated aging phenotype, with a shorter lifespan and earlier loss of fertility [24]. Both Ames and Snell mice show increased insulin sensitivity [25,26]. 

Another pituitary mutation that causes dwarfism was found in *lit/lit* mice. The responsive gene in these mice is the GH-releasing hormone receptor (*Ghrhr*) [27]. *Ghrhr^lit/lit^* mice have low levels of GH, IGF-I with prolonged lifespan [28]. Data from the *Ghrhr^lit/lit^* mice also suggest that changes in the GH/IGF-I axis could be sufficient to extend lifespan of dwarf mice, because these mice have normal PRL, TSH and thyroid hormone levels. *Ghrhr^lit/lit^* mice appear to have normal insulin responses compared to wild type mice [29]. However, double mutant mice of *Ghrhr *and PRL-receptor showed decreased insulin sensitivity in old age because of increased adiposity [29]. This contrasts with the results of the GH- and PRL-deficient Ames and Snell mice described above. 

Pituitary specific GH-antisense TG rats show increase lifespan with lower serum insulin, IGF-I compared to Wistar rats, which have same genetic background [14,15]. These rats have increased insulin action to reduce blood glucose; however, insulin-independent glucose-lowering effects have also been suggested [30,31]. These rats show a moderate reduction of the GH/IGF-I axis compared to Ames or Snell dwarf mice whose GH/IGF-I axis is almost completely suppressed. Similar to these dwarf mice, CR upon GH-antisense TG rats have an extended lifespan with further reduction of insulin/IGF-I levels [14]. 

The murine equivalents to human Laron syndrome are GH receptor/binding protein homozygous knockout mice (*GHR/BP^-/-^*) [32]. The human and murine *GHR/BP* gene encodes for two proteins; GH receptor and GH receptor binding protein, a truncated form of a receptor. GHR/BP KO mice have a longer lifespan compared to control wild type mice [33]. These mice have a markedly reduced plasma concentration of IGF-I, and a dwarf phenotype with increased insulin sensitivity [33,34]. GHR/BP deficiency impairs the beneficial effects of CR on lifespan extension and increases insulin sensitivity [35]. As well, CR increases insulin sensitivity in control mice, but does not further increase insulin sensitivity in GHR/BP^-/-^ mice [35].

In the knockout mice heterozygous for the IGF-I receptor (*Igf1r^**+/-**^*), significant lifespan extension was observed in female mice, whereas homozygous knockout mice died at birth, probably as a result of respiratory failure [36]. The body size of these mice is almost normal and they have no detectable alterations in reproductive development and function. Male *Igf1r^**+/-**^* mice show reduced insulin sensitivity, whereas females showed increased insulin sensitivity [36]. Mouse embryonic fibroblasts (MEFs) from *Igf1r^**+/-**^* mice show reduced IGF-I receptor (IGF-IR) signaling in response to IGF-I addition compared to MEFs from wild type mice [36]. Namely, tyrosine phosphorylation levels of IGF-IR, IRS-1, and p66^shc^ proteins are decreased in MEFs from *Igf1r^**+/-**^* mice. Moreover, it has been documented that brain specific heterozygous IGF-IR knockout mice have an increased lifespan and a significant decrease in mortality rate [37]. This is interesting because importance of brain insulin/IGF-I signaling is also suggested in *C. elegans* and *Drosophila* [37].

Fat specific insulin receptor knock out mice (FIRKO mice) show increased lifespan without reduction of the plasma IGF-I levels [38,39]. These mice show reduced body fat content, body mass index, and resistant to age-related and hyperphagia-induced insulin resistance. In adipose tissue, insulin-stimulated glucose uptake was reduced in FIRKO mice, indicating that insulin signaling is markedly decreased in the adipose tissue of these mice. However, whole body insulin sensitivity is increased in FIRKO mice probably as a result of the increased plasma adiponectin levels, especially in aged mice [38,39]. Recent study has revealed that FIRKO mice maintain high mitochondrial capacity and metabolic rates in adipose tissue [40]. These increased mitochondrial functions may be important for the lifespan extension seen in the FIRKO mouse.

Proto-oncogene Shc, an adaptor protein of receptor tyrosine kinase, is phosphorylated by the activation of growth factor receptors such as IGF-IR and insulin receptor (IR); a knock out mouse strain of the p66 isoform of Shc, p66^shc^ showed a prolonged lifespan and increased oxidative stress resistance [41]. The mammalian *Shc* locus encodes three protein isoforms; p46^shc^, p54^shc^, and p66^shc^ [42]. MEFs from p66^shc^ knock out mice show more resistance to hydrogen peroxide treatment or UV radiation compared to MEFs from wild type mice [41]. This effect partly is mediated the induction of FoxO3a, a mammalian homologue of the *C. elegans *forkhead transcription factor *daf-16* [43]. This is interesting because *daf-16* is essential for lifespan extension in the *daf-2* mutant [44]. No information is yet available about the insulin sensitivity of p66^shc-/-^ mice.

IRS-1 knock out (*Irs1^-/-^*) female mice show increased lifespan despite moderate insulin resistance [45]. Same as *Igf1r^**+/-**^* mice, *Irs1^-/-^*mice lifespan extension is predominantly seen in females. The body weight of these mice is lower than that of wild type; however, serum IGF-I levels are not different. These mice show reduced adiposity similar to CR and FIRKO mice; however, *Irs1^-/-^*  mice show moderate insulin resistance. Thus, the improvement in insulin sensitivity associated with reduced fat mass is not a prerequisite for the lifespan extension of *Irs^-/-^* mice [45]. Tyrosine phosphorylation of IRS-1 enhances insulin sensitivity, whereas serine phosphorylation of IRS-1 by S6 kinase induces insulin resistance [46]. Therefore, IRS-1 has both negative and positive effects on insulin/IGF-I signaling. Lack of IRS-1 might cause predominately favorable intracellular signaling situations for cell survival causing lifespan extension through the activation of the FoxO1 protein, a forkhead transcription factor important for oxidative stress response.

IRS-2 heterozygous knock out mice, specifically in brain (*bIrs2^**+/-**^*), showed increased lifespan [47]. Interestingly, while systemic heterozygous IRS-2 deficiency improved insulin sensitivity, brain-specific IRS-2 knock out led to insulin resistance. Although the mechanism responsible for this discrepancy is unknown, these results also support that insulin sensitivity by itself is not a prerequisite for lifespan extension in insulin/GH/IGF-I signal modified longevity rodents. Similar to IRS-1, tyrosine phosphorylation of IRS-2 by IR or IGF-IR is the first event in the activation of the intracellular insulin/IGF-I signal transduction pathway. IRS-1 and IRS-2 are the major IRS leading to glucose homeostasis, and have distinct and overlapping roles in diverse organs [48]. Lifespan extension in *bIrs2^**+/-**^* mice might be related to the FoxO1 protein levels maintained in the brain that lead to the expression of antioxidant protein SOD2 [47]. These results of lifespan extension of *bIrs2^**+/-**^* as well as *Irs1^-/- ^*mice might suggest that the mutation of particular molecules of intracellular insulin/IGF-I signaling is sufficient to induce longevity. Therefore, a reduced GH/IGF-I axis in dwarf animals and long-lived insulin/GH/IGF-I receptor modified mice might eventually change the activation of these intracellular substrate molecules including p66^shc^ and their downstream targets for lifespan extension. Hence, reduced body size and increased insulin sensitivity might not be important and so could be separated from factors responsible for lifespan extension in such animals.

In summary, the above mentioned models suggest that decreased body weight and increased insulin sensitivity might be not prerequisites for lifespan extension in insulin/GH/IGF-I modified longevity rodents. Although there a significant effect on body weight and insulin sensitivity were seen depending on the particular factors in the breeding environments used in the study, such as diet composition, the results presented highlight that the importance of the intracellular signaling molecules of the insulin/GH/IGF-I signal rather than the upstream hormonal signals. Fig. (**1**) shows the sites of mutations in rodent longevity models. Pituitary GH and insulin/GH/IGF-I receptor mutations might cause favorable situations for intracellular signaling molecules, such as reduction of function IRS-1, -2 and p66^shc^, because knock out of these intracellular molecules extends lifespan. Recently, we isolated WDR6, a novel candidate for the intracellular insulin/IGF-I signaling molecule. Next we discuss the potential involvement of WDR6 in lifespan regulation. 

### WDR6 as a New Candidate for an Insulin/GH/IGF-I Signaling Molecule Related to Lifespan Regulation

We recently isolated and identified WDR6 as a novel molecule involved in hypothalamic insulin/IGF-I signaling with interacting IRS-4 by cDNA subrtaction method [22, 49]. IRS-1 and IRS-2 are ubiquitously expressed, while IRS-3 and IRS-4 are tissue specific, detected in significant amounts in fat and brain tissue, respectively [50]. IRS-4 is expressed abundantly in the arcuate nucleus, an important hypothalamic nucleus for regulation of energy and metabolism [51]. We found that the expression of WDR6 was decreased *in vivo* upon CR as well as with suppression of the GH/IGF-I axis; both treatments cause reductions of plasma insulin/IGF-I concentrations. Our *in vitro* study indicated that the addition of insulin or IGF-I caused increased gene expression of WDR6 in GT1-7 cells, a mouse hypothalamus derived cell line [22,52]. These results suggested the presence of a feedback loop for insulin/IGF-I-mediated expression of WDR6.

WD repeats are conserved domains of approximately 40 to 60 amino acids that contain a tryptophan-aspartic acid (WD) dipeptide at their C-terminus, and have a conserved core sequence [53,54]. WDR6 was first cloned by Li *et al*, [55]. They showed that human WDR6 has 11 WD-repeat domains and shows relatively ubiquitous expression. However, we isolated the WDR6 gene in brain as a hypothalamus enriched gene [22] by using a cDNA subtraction method [49,56,57]. This expression pattern of WDR6 is consistent with another report that used a DNA microarray analysis [58]. WD repeat domains are involved in mediating protein-protein interactions [59,60], and WD repeat-containing proteins have a central role in many signal transduction cascades by coordinating the interaction of key signaling molecules. 

We found that IRS-4 interacts with WDR6 *in vivo*. Our results suggested that WDR6 might regulate phosphorylation levels of the interacting protein, IRS-4 [22]. Interestingly, it was suggested that WDR6 and IRS-4 might also be involved in fibroblast growth factor (FGF) signaling [61]. Stimulation with FGF2 induced a 3-fold increase in tyrosine phosphorylated proteins, as assessed by mass spectrometry [61]. It has been suggested that FGF2 has an inhibitory effect on food intake and locomotor activity in rats [62]. Taken together, these results suggest that WDR6 may be involved in feeding and physical activities through its interaction in the brain with IRS-4.

Mice lacking IRS-4 show mild defects in growth, reproduction and glucose homeostasis [63]. These mice show slightly impaired responses on the oral glucose tolerance test. The insulin tolerance testing also showed these mice slightly less responsive to insulin compared to wild type; however, the effect was statistically insignificant. It was suggested that loss-of-function of IRS-4 in the brain might cause these phenotypes, because IRS-4 is predominantly expressed in the brain [63]. These phenotypes are somewhat similar to long-lived *Irs1^-/-^* and *bIrs2^**+/-**^* mice. Study is needed to find out whether WDR6 also interacts with IRS-1 and IRS-2. 

It has recently been shown that WDR6 interacts with LKB1, a tumor suppressor gene encoding a serine/threonine kinase that phosphorylates AMP-activated protein kinase (AMPK), and regulates p27^Kip1^ induction [64]. AMPK is a critical component in a protein kinase cascade that have a crucial role in the regulation of energy balance, particularly in the brain [65,66]. LKB1 gene expression is enriched in the hypothalamus, similar to the WDR6 gene in the brain [58]. The LKB1-AMPK-p27 pathway is also involved in the autophagic response, a bulk misfolded protein degradative system [67]. In *C. elegans*, autophagy genes are essential for dauer development and for lifespan extension in the *daf-2* mutant [68]. Moreover, autophagy mediates lifespan extension of *C. elegans* upon CR [69]. Autophagy is also reported to be important in lifespan extension in *Drosophila* [70]. In mammals, the induction of autophagy is a protective response against neurodegenerative diseases [71,72]. Moreover, CR prevents the age-dependent decline of autophagic proteolysis in liver cells [73]. Thus, it is interesting to speculate that WDR6 also regulates autophagy responses by modulating the LKB1-AMPK pathway in the brain. Further studies using genetically modified mice such as conditional knock out varieties, should help gain an understanding of the precise mechanism of WDR6 involvement in AMPK signaling as well as in insulin/IGF-I signaling in longevity.

In conclusion, increased insulin sensitivity might not be a prerequisite for lifespan extension in insulin/GH/IGF-I modified rodent longevity models. Although it is possible that different tissues show different insulin sensitivity in these longevity models, there could be more important intracellular signaling molecules in insulin/GH/IGF-I signaling, such as IRS-1, -2, and p66^shc^, and possibly WDR6, for lifespan extension. These molecules could be points of intervention for therapies with potential to delay the pathogenesis of age-related diseases and even the aging process. Further study is needed to reveal the importance, if any, of these molecules through identification of target transcription factors critical for lifespan extension.

## Figures and Tables

**Fig. (1):**
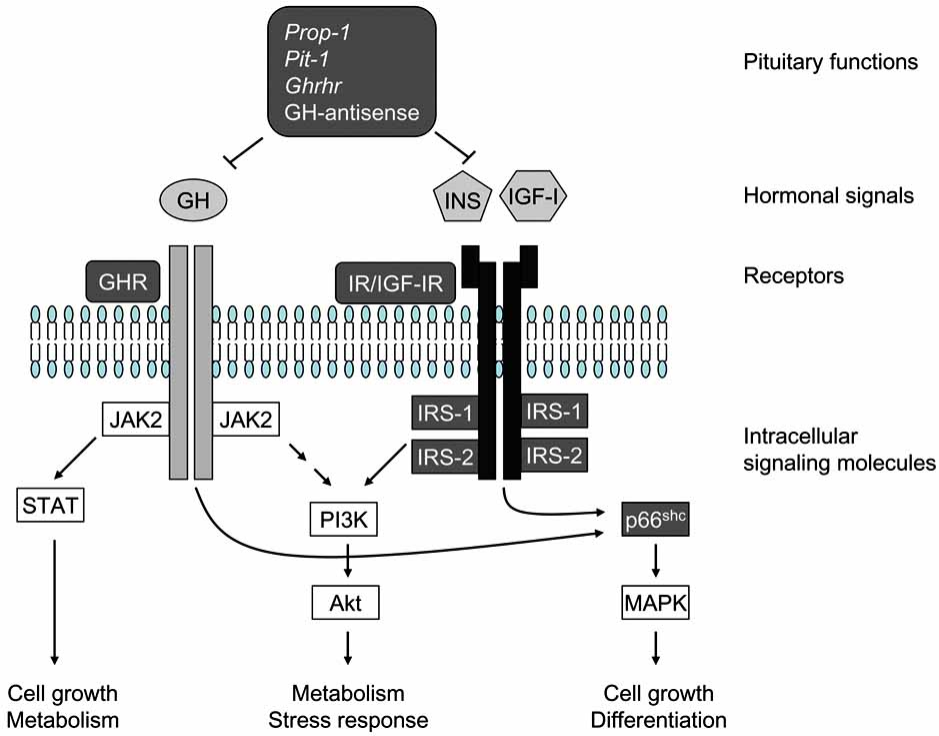
Mammalian GH and insulin/IGF-I signaling pathways. Longevity related genes shown in Table **1** and discussed in text are shown in outlined characters on a gray background. Pituitary mutations cause GH/IGF-I-deficiency results in dwarf phenotypes, and increased insulin sensitivity in most cases. Mutations of receptors and intracellular signaling molecules are not necessary to increase insulin sensitivity for lifespan extension. These intracellular molecules could be points of intervention for therapies with the potential to delay the aging process.

**Table 1.** Rodent Longevity Models of Modified Insulin/GH/IGF-I Signaling

Rodent Models	Responsive Gene	Effect^a^	Lifespan^b^	Body Weight^c^	IGF-I	Insulin Sensitivity
Ames mice	*Prop-1*	Pituitary	♂49%,♀68%	33%	Decreased	Increased
Snell mice	*Pit-1*	Pituitary	♂26%,♀42%	33%	Decreased	Increased
*Ghrhr^lit/lit^*mice	*Ghrhr*	Pituitary	♂23%,♀25%	67%	Decreased	Unchanged
GH-antisense TG rats	GH-antisense	Pituitary	♂10%	66%	Decreased	Increased
*GHR/BP^-/-^* mice	*GHR/BP*	Receptor	♂55%,♀38%	40-41%	Decreased	Increased
*Igf1r^+/-^*mice	*Igf1r*	Receptor	♀33%	92-94%	Increased	Increased
FIRKO mice	*IR *(fat-specific)	Receptor	♂+♀18%	75-85%	Unchanged	Increased
*p66^shc-/-^*mice	*p66^shc^*	Intracellular	♂+♀30%	100%	Unknown	Unknown
*Irs1^-/-^*mice	*Irs1*	Intracellular	♀32%	60-65%	Unchanged	Decreased
*bIrs2^+/-^*mice	*Irs2 *(brain-specific)	Intracellular	♂18%	95-100%	Unknown	Decreased

a Site of primary effect of the mutation.

b % increase of lifespan in mutants compared to control animals.

c % of body weight compared to control animals (6 to 10 months old).
